# An error-resilient non-volatile magneto-elastic universal logic gate with ultralow energy-delay product

**DOI:** 10.1038/srep07553

**Published:** 2014-12-23

**Authors:** Ayan K. Biswas, Jayasimha Atulasimha, Supriyo Bandyopadhyay

**Affiliations:** 1 Department of Electrical and Computer Engineering, Virginia Commonwealth University, Richmond, VA 23284, USA; 2 Department of Mechanical and Nuclear Engineering, Virginia Commonwealth University, Richmond, VA 23284, USA

## Abstract

A long-standing goal of computer technology is to process and store digital information with the *same* device in order to implement new architectures. One way to accomplish this is to use nanomagnetic logic gates that can perform Boolean operations and then store the output data in the magnetization states of nanomagnets, thereby doubling as both logic and memory. Unfortunately, many of these nanomagnetic devices do not possess the seven essential characteristics of a Boolean logic gate : concatenability, non-linearity, isolation between input and output, gain, universal logic implementation, scalability and error resilience. More importantly, their energy-delay products and error rates tend to vastly exceed that of conventional transistor-based logic gates, which is unacceptable. Here, we propose a non-volatile voltage-controlled nanomagnetic logic gate that possesses all the necessary characteristics of a logic gate and whose energy-delay product is two orders of magnitude *less* than that of other nanomagnetic (non-volatile) logic gates. The error rate is also superior.

There is significant interest in ‘non-volatile logic’ because the ability to store and process information with the same device affords immense flexibility in designing computing architectures. Non-volatile logic based architectures can reduce overall energy dissipation by eliminating refresh clock cycles, improve system reliability and produce ‘instant-on’ computers with virtually no boot delay. A number of non-volatile universal logic gates have been proposed to date^1^,^2^,^3^, but they do not necessarily satisfy all the requirements for a logic gate^4^,^5^ and therefore may not be usable in all circumstances. Ref. 1 proposed an idea where digital bits are stored in the magnetization orientations of an array of dipole-coupled nanomagnets and dipole coupling between neighbors elicits logic operation on the bits. This gate is not concatenable since the input and output bits are encoded in dissimilar physical quantities: the inputs are encoded in directions of magnetic fields and the output is encoded in the magnetization orientation of a magnet. Thus, the output of a preceding gate cannot act as the input to the succeeding gate without additional transducer hardware to convert the magnetization orientation of a nanomagnet into the direction of a magnetic field. The gate also lacks true gain since the energy needed to switch the output comes from the inputs and not an independent source such as a power supply. Additionally, the strength of dipole coupling between magnets decreases as the square of the magnet's volume, which limits scalability. Finally, dipole coupling is not sufficiently resilient against thermal noise, resulting in unacceptably large dynamic bit error probability in dipole-coupled logic gates^6^,^7^,^8^.

Ref. 2 proposed a different construct where a NAND gate was implemented with a single magneto-tunneling junction (MTJ) placed close to four current lines, two of which ferry the two input bits to the gate, the third is required for an initialization operation, and the fourth carries the output. The magnetic fields generated by the input currents iflip the magnetization of the MTJ's soft layer and switch its resistance, thereby switching the magnitude of the output current and performing NAND logic operation. Slightly different renditions of this idea have been proposed^9^ and an experimental demonstration has been reported^10^. Unfortunately, this gate too is not directly concatenable since the input bits are encoded in the *directions* of the input currents while the output bit is encoded in the *magnitude* of the output current. Moreover, since it is difficult to confine magnetic fields to small regions, the separation between neighboring devices must be large. Individual devices can be small in size, but because the inter-device pitch is large, the device density will be small. There is also some chance that the output current can, by itself, switch the magnetization of the magnetic layers and therefore affect its own state. This is equivalent to lack of isolation between the input and the output, which makes gate operation unreliable. Finally, another MTJ-based logic gate was recently proposed^11^, but it requires a feedback circuit to operate (which makes it extremely energy-inefficient and error-prone) and, additionally, the design is flawed^12^. Thus, while these devices are interesting in their own right, they may not be universally usable.

A more recent scheme that overcomes most of the above shortcomings was proposed in Ref. 3, 4. It implements non-volatile logic with nanomagnets switched by spin currents. Both computation and communication between gates are carried out with a sequence of clock pulses. Unfortunately, its error-resilience has not been examined. Normally, magnetic devices are much more error-prone than transistors since magnetization dynamics is easily disrupted by thermal noise^6^,^7^. Logic has stringent requirements on error rates and it is imperative to evaluate the dynamic bit error probability of any gate to assess its viability.

Finally, the most important metric for a logic gate is the energy-delay cost. All non-volatile magnetic logic schemes fail in this area. The scheme in Ref. 2 uses current-generated magnetic fields to switch magnets and hence would dissipate at least 10^9^ kT of energy per gate operation at room temperature to switch in ~1 ns^13^ (energy-delay product = 4 × 10^−21^ J-s). A recent experiment conducted to demonstrate this scheme used on-chip current-generated magnetic fields to switch magnets and ended up dissipating approximately 10^12^ kT of energy per switching event, despite switching in ~1 *µ*s (energy-delay product = 4 × 10^−15^ J-s)^14^. The scheme in Ref. 3 is expected to dissipate between 10^5^ and 10^6^ kT of energy when it switches in 1 ns (energy-delay product = 4 × 10^−25^ - 4 × 10^−24^ J-s)^15^, although a lower energy-delay product may be possible with design optimization [16]. In contrast, a low-power transistor may dissipate only 10^3^ kT of energy when it switches in 0.1 ns (energy-delay product = 3 × 10^−28^ J-s)^17^. Therefore, all the above non-volatile schemes appear to be far inferior to transistors in energy-delay product, which may preclude their widespread application, despite the non-volatility.

In this report, we propose a non-volatile nanomagnetic NAND gate that is switched with voltage (not current) unlike the other schemes. It has an energy-delay product of 1.6 × 10^−26^ J-s, which is smaller than that of other magnetic logic schemes by approximately on order of magnitude. The energy-delay product, however, by itself, is not the most meaningful metric for benchmarking device performance. It is always possible to reduce this product arbitrarily by sacrificing reliability. For example, one can forcibly switch a device faster and also dissipate less energy to switch (which will reduce the energy-delay product), but at the expense of increased switching failures. A more meaningful metric may be the product of energy, delay and failure (error) probability. The error probability of the proposed logic gate has been evaluated rigorously (with stochastic simulation) to establish its reliability. With careful choice of parameters, it is possible to reduce the gate error probability to below 10^−8^ at room temperature, which is remarkable for magnetic logic. Finally, the proposed gate fulfills all the requirements for logic. Therefore, it is the first nanomagnetic logic gate that has the cherished advantage of magnetic logic gates (non-volatility) and yet none of the usual disadvantages.

The proposed gate structure is shown in Fig. 1(a). It is implemented with a skewed MTJ stack, resistors *R*, a bias dc voltage *V_BIAS_*, and a constant current source *I_BIAS_*. The current source is not used to switch the gate, but merely to produce an output voltage *V_out_* representing the output logic bit. Input bits are encoded in input voltages *V_in_*. Both input and output bits are encoded in the same physical quantity, voltage, which allows direct concatenation.

The bottom layer of the MTJ stack is an elliptical magnetostrictive (metallic) nanomagnet (Terfenol-D) and the top layer is a non-magnetostrictive elliptical (metallic) synthetic anti-ferromagnet (SAF) with large shape anisotropy. The top layer acts as the hard (or pinned) layer and the bottom layer acts as the soft (or free) layer of the MTJ. There is a small permanent magnetic field directed along the minor axis of the magnetostrictive nanomagnet (+y-direction) which brings its two stable magnetization orientations out of the major axes and aligns them along two mutually perpendicular in-plane directions that lie between the major and minor axes (Fig. 1(b))^18^,^19^. The major axis of the top SAF layer is aligned along one of the two stable magnetization orientations of the soft magnet. It is then permanently magnetized in the direction *anti-parallel* to that orientation. Two electrodes *E* and *E*′ are delineated on the PZT surface such that the line joining their centers lies close to that orientation. The electrode lateral dimensions, the separation between their edges, and the PZT film thickness are all approximately equal.

The two electrodes *E* and *E*′ are electrically shorted. Whenever an electrostatic potential difference appears between them and the conducting silicon substrate (between point-*M* and point-*N* in Fig. 1(a)), the PZT layer is strained. Since the electrode in-plane dimensions are comparable to the PZT film thickness, the out-of-plane (*d*_33_) expansion/contraction and the in-plane (*d*_31_) contraction/expansion of the piezoelectric regions underneath the electrodes produce a highly localized strain field under the electrodes^20^. Furthermore, since the electrodes are separated by a distance approximately equal to the PZT film thickness, the interaction between the local strain fields below the electrodes will lead to a biaxial strain in the PZT layer underneath the soft magnet^20^. This biaxial strain (compression/tension along the line joining the electrodes and tension/compression along the perpendicular axis) is transferred to the soft magnetostrictive magnet in elastic contact with the PZT, thus rotating its magnetization via the Villari effect. This happens despite any substrate clamping and despite the fact that the electric field in the PZT layer just below the magnet is approximately zero^20^. Some of the generated strain may even reach the top hard magnet^21^, but since the hard magnet is very anisotropic in shape and is not magnetostrictive, its magnetization will not rotate perceptibly. Rotation of the magnetization of the soft layer of an MTJ due to strain has been recently demonstrated experimentally^21^.

Fig. 2 shows the potential energy profile of the soft magnetostrictive nanomagnet in its own plane (*ϕ* = 90°) plotted as a function of the angle *θ* subtended by the magnetization vector with the major axis of the ellipse (z-axis). Note that the energy profile has two degenerate minima (*B* and *C*) in the absence of stress (i.e. when no voltage is applied between nodes *M* and *N*). These two states correspond to Ψ_1_ and Ψ_0_, respectively, in Fig. 1. Application of sufficient potential difference between *M* and *N*, to generate sufficient stress in the magnetostrictive magnet, transforms the energy profile into a monostable well (with no local minima) located at either *B* or *D*, depending on whether the stress is tensile or compressive, i.e. whether node *M* is at a higher potential than node *N*, or the opposite^18^,^19^. If we apply compressive stress with the right voltage polarity, the system will go to point *D* and the magnetization will point along the corresponding direction. Thereafter, if we withdraw the voltage and stress, the system will go to the *nearer* energy minimum at point *C* (and not the other minimum at *B*) because of the potential barrier that exists between *B* and *C*. This happens with >99.999999% probability at room temperature in the presence of thermal noise (see supplementary material). Once it reaches *C*, the system will remain there (since it is an energy minimum) and the magnetization will continue to point along the corresponding direction (making the device non-volatile) until tensile stress is applied [by applying voltage of opposite polarity between *M* and *N*] to take the system to *B*, thereby changing the magnetization to the other stable direction. Upon withdrawal of the tensile stress, the system will remain in state *B* because the energy barrier between *B* and *C* will prevent it from migrating to *C*. Therefore, the system is non-volatile in either state. By merely choosing the *polarity* of the voltage between nodes *M* and *N*, we can deterministically visit either state *B* or state *C* and orient the magnetization along either of the two stable states. The magnet will remain in the chosen state after the voltage is withdrawn. This was used as the basis for deterministically writing the bit 0 or 1 in non-volatile memory, irrespective of what the initial stored bit was^18^,^19^. Here, we have extended that idea to build a non-volatile universal logic gate (NAND) using a magneto-tunneling junction in the manner of Ref. 2.

The gate works as follows: Let us first assume that the binary logic bits ‘1’ and ‘0’ are encoded in voltage levels *V*_0_ and *V*_0_/2 [what determines the minimum value of *V*_0_ is discussed later]. The bias voltage is set to *V_BIAS_* = 5*V*_0_/12. Every logic operation is preceded by a RESET operation where the two inputs *V_in_*_1_ and *V_in_*_2_ are set to *V*_0_/4. During RESET, the potential drop appearing between the terminals *M* and *N* in Fig. 1 is *V_MN_* = −*V*_0_/4, which generates in-plane tensile stress in the direction of the line joining the two electrodes and in-plane compressive stress in the direction perpendicular to the line joining the two electrodes. This moves the system to point *B* in the energy profile in Fig. 2 where the magnetization vector is nearly anti-parallel to the magnetization of the top magnet (SAF) [see the state ‘Ψ_1_’ in Fig. 1(b)]. This makes the resistance of the MTJ ‘high’. When the input voltages are subsequently withdrawn by grounding the inputs and shorting the bias voltage source connected to the Si substrate, *V_MN_* drops to nearly zero as long as *R* is much greater than the resistance of the ultrathin PZT layer. Therefore, the stress in the magnet relaxes, but the system remains at point *B*. Consequently, the MTJ is always left in the high resistance state after the RESET step is completed.

In the logic operation stage, the following scenarios occur: (1) if both inputs are low (i.e. *V_in_*_1_ = *V_in_*_2_ = *V*_0_/2), then *V_MN_* = −*V*_0_/12; (2) if either input is low (i.e. *V_in_*_1_ = *V*_0_ and *V_in_*_2_ = *V*_0_/2, or vice versa), then *V_MN_* = *V*_0_/12 (see supplementary material). The potential energy profiles for these two scenarios are shown in Fig. 3. When both inputs are low, the global energy minimum is at *B*′ ≈ *B*. Since the RESET operation left the system at *B*, the magnetization barely rotates and the MTJ resistance remains high. When one input is high and the other low, the *global* energy minimum moves to *B*″ which is closer to the other stable magnetization orientation, but there is still a *local* energy minimum close to *B* which is separated from *B*″ by a potential barrier that *cannot be crossed*. Therefore, the system remains stuck in the metastable state corresponding to the local minimum near *B* and the magnetization does not rotate perceptibly. Hence, once again, the MTJ resistance remains high. After the inputs are removed by grounding *V_in_*_1_ and *V_in_*_2_, shorting the bias voltage sources, and open-circuiting the bias current source, the strain in the magnet relaxes and the magnetization settles into the only accessible stable state *B*. It remains there in perpetuity thereby implementing *non-volatile* logic (memory of the last output state is retained). However, (3) if both inputs are high, then *V_MN_* = +*V*_0_/4 (see supplementary material) and the strain becomes in-plane compressive in the direction of the line joining the two electrodes and in-plane tensile in the direction perpendicular to the line joining the two electrodes. This is sufficient to change the potential energy profile dramatically as shown in Fig. 3. Now the operating point moves to *D*′ since it becomes the global minimum and there is no local minimum where the system can get stuck. Consequently, the magnetization vector rotates to an orientation nearly perpendicular to the magnetization of the top layer [state ‘Ψ_0_’ in Fig. 1(b)]. The resistance of the MTJ then drops by ~50% since the resistance is approximately proportional to *cos*^2^(*γ*/2), where *γ* is the angle between the magnetizations of the top and bottom magnets, assuming that the spin injection and detection efficiencies of the magnet-spacer interfaces are ~100%^22^ [if the efficiencies are less than 100%, the logic levels will be encoded in *V*_0_ and *xV*_0_, where *x* > 0.5]. Subsequent removal of the input voltages (by grounding them shorting the bias voltage sources), drives the system to state *C* where the MTJ resistance remains low, thereby retaining memory of the last output state (non-volatility). The probability of the gate working in this fashion, in the presence of thermal noise, has been calculated rigorously from stochastic Landau-Lifshitz-Gilbert simulations of the magnetodynamics (see supplementary material) and that probability was found to exceed 99.999999% in all cases.

Let us now explain how this translates to NAND logic. Since there is not much electric field in the PZT directly under the MTJ stack^20^, we can neglect any voltage drop in the PZT between the magnetostrictive magnet and the silicon substrate. Therefore, *V_out_* = *I_BIAS_R_MTJ_*, where *R_MTJ_* is the resistance of the MTJ stack. The biasing constant current source *I_BIAS_* is set to *V*_0_/*R_high_*, where *R_high_* is the resistance of the MTJ in the high-resistance state. Therefore, whenever the MTJ is in the high resistance state, the output voltage is *V*_0_ and whenever the MTJ is in the low resistance state, the output voltage is *I_BIAS_R_low_* = *V*_0_/2 because *R_low_* = *R_high_*/2 [*R_low_* is the resistance of the MTJ in the low resistance state]. Since the logic bit 1 is encoded in voltage *V*_0_ and logic bit 0 is encoded in the voltage level *V*_0_/2, we find that the output bit is 1 when either input bit is 0, and it is 0 when both inputs are 1. In other words, we have successfully implemented a NAND gate (see the truth table shown in Fig. 1).

Let us now examine if this device fulfills all the requirements of a Boolean logic gate.

*a. Concatenability:* For concatenability, the output voltage of a preceding gate has to be fed directly to the input of a succeeding gate. This requires that *V_in_*_1_(*high*) = *V_in_*_2_(*high*) = *I_BIAS_R_high_* = *V*_0_, and *V_in_*_1_(*low*) = *V_in_*_2_(*low*) = *I_BIAS_R_low_* = *V*_0_/2 which is easily achieved by choosing *I_BIAS_* = *V*_0_/*R_high_*. In the event the logic levels have to be en-coded in *V*_0_ and *xV*_0_ (0.5 ≤ *x* ≤ 1), the resistive network at the input side and *V_BIAS_* have to be re-designed, but this is trivial.

*b. Non-linearity:* Since the MTJ resistance has only two values (high and low), the gate is inherently non-linear^3^.

*c. Isolation between input and output:* The output voltage cannot change the input voltage levels in any way. This results in isolation.

*d. Gain:* Gain is ensured when the energy to switch the output bit does not come from the input energy, but from an independent power source^3^, which, in our case, is the constant current source. Whenever the inputs *V_in_*_1_ and *V_in_*_2_ end up switching the MTJ resistance, the independent current source *I_BIAS_* switches *V_out_*.

*e. Universal logic:* The gate performs NAND operation which is universal.

*f. Scalability:* Because we do not use magnetic fields to switch specific gates (unlike refs. 1, 2), but instead use only voltages, we do not have to space gates far apart so that fringing magnetic fields from one gate do not inuence the neighbor. As a result, gates can be placed close to each other, thereby increasing the gate density. The gates can scale all the way down to the superparamagnetic limit of the nanomagnets at the operating temperature.

*g. Error-resilience:* Two types of errors afflict non-volatile gate operation: *static* errors caused by the magnetization of the soft magnetostrictive layer flipping spontaneously owing to thermal noise [thereby switching the output bit erroneously in standby state], and *dynamic* errors that occur (also because of thermal noise) when the output switches to an incorrect state in response to the inputs changing. The static error probability is determined by the energy barrier separating the two stable magnetization states in the soft layer. The minimum barrier height is determined by the magnetic field strength, the dimensions of the magnet and material parameters. In our case, it was 69.26 kT at room temperature (see supplementary material), so the static error probability is ~ *e*^−69.26^ ≈ 10^−30^ per spontaneous switching attempt^23^. In other words, the retention time of an output bit in the non-volatile logic gate at room temperature will be ~ (1/*f*_0_) *e*^69.26^ = 3.8 × 10^10^ years, since the attempt frequency *f*_0_ in nanomagnets will very rarely exceed 1 THz^24^. In other words, the gate is indeed non-volatile. Dynamic gate errors, however, are much more probable and accrue from two sources: (1) thermal noise causing erratic magnetization dynamics that drive magnets to the wrong stable magnetization state resulting in bit error, and (2) complicated clocking schemes that require precise timing syn-chronization for gate operation and whose failure cause bit errors. The gate in ref. 3, which is the only other nanomagnetic gate known to us that fulfills nearly all the requirements of logic, works with Bennett clocking^25^ which is predicated on the principle of placing the output magnet in its maximum energy state, and then waiting for the input signal to drive it to the desired one among its two minimum energy states to produce the correct output bit. This strategy is risky since the maximum energy state is also maximally unstable. While perched on the energy maximum, thermal uctuations can drive the output magnet to the wrong minimum energy state with unacceptably high probability^6^, resulting in unacceptable bit error rates. A later modification^4^ overcame this shortcoming, but at the expense of much increased energy dissipation. Moreover, that logic gate also requires a complicated clocking sequence without which it cannot operate. In contrast, we *never* place any element of our gate at the maximum energy state (no Bennett clocking) and no complicated clocking sequence is needed.

An important consideration for Boolean logic is *logic level restoration*^26^. If noise broadens the input voltage levels *V*_0_ and *V*_0_/2, making it harder to distinguish between bits 0 and 1, the logic device should be able to restore the distinguishability by ensuring that the output voltage levels are not broadened and remain well separated. For this, the transfer characteristic of the gate (when used as an inverter) must show a sharp transition. We have computed the transfer characteristic (*V_out_* versus *V_in_*) by shorting the inputs and calculating the output *V_out_* for various values of *V_in_*. The calculation procedure in described in the supplementary section. The characteristic is shown in Fig. 4 and the sharpness of the transition allows for excellent logic level restoration capability.

The proposed gate has unprecedented energy-efficiency that far exceeds that of other non-volatile magnetic NAND gates. There are four contributions to the energy dissipated in this logic gate during a logic operation: internal dissipation due to Gilbert damping that occurs while the magnetostrictive layer's magnetization switches (rotates), energy *C*(*V_MN_*)^2^ dissipated in turning on/off the potential *V_MN_* = ±*V*_0_/4 ( = 112.5 mV) abruptly or non-adiabatically during the RESET stage or logic operation stage (where *C* is the capacitance between the shorted pair of electrodes and the n^+^-Si substrate), the energies dissipated in the resistors *R*, and the maximum energy 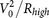 dissipated in the MTJ when the output is high (the energy dissipated when the output is low is 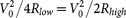, which is 50% lower). We can make the energies dissipated in the resistors arbitrarily small by choosing arbitrarily high values for *R*; hence, this contribution is neglected. The other contributions are computed in the supplementary material and add up to a mere 3004 kT (12.5 aJ) at room temperature. This dissipation is comparable to that of state-of-the-art low-power complementary-metal-oxide-semiconductor transistor (CMOS) based two-input NAND gates^17^. The switching time, on the other hand, is ~1.3 ns, which is one order of magnitude longer than that of the CMOS based logic gate. However, the CMOS based gate is volatile while this gate is non-volatile. The overall energy delay product of this gate (1.6 × 10^−26^ J-s) is about two orders of magnitude superior to that of any other magnetic (non-volatile) logic gate^17^.

Logic gates of this type may have a special niche for medically implanted processors such as pacemakers^27^, wearable electronics^28^, or devices implanted in an epileptic patient's brain that monitor brain signals and warn of an impending seizure. They also need to dissipate very little energy so that they can be powered by the energy harvested from the user's body movements and not require a battery^29^. The present device is tailor-made for such applications.

## Methods

To fabricate the gate, a piezoelectric (PZT) thin film (~100 nm thick) is deposited on a conducting n^+^-Silicon substrate which is grounded through a bias voltage *V_BIAS_*. A skewed MTJ stack is fabricated on top of the PZT film. The bottom layer material is chosen as Terfenol-D because of its large magnetostriction (900 ppm). The magnetostriction is positive which tends to make the magnetization align along the direction of tensile stress and perpendicular to the direction of compressive stress. The angle between the major axes of the two elliptical nanomagnets is determined by the angular separation between Ψ_1_ and Ψ_0_. The current source *I_BIAS_* is connected across the MTJ stack. The magnetostrictive nanomagnet has a major axis of 100 nm, minor axis of 42 nm and thickness of 16.5 nm, which ensures that it has a single ferromagnetic domain.

To evaluate the dynamic error probability, the magnetization dynamics of the soft magne-tostrictive magnet induced by stress in the presence of thermal noise is modeled by the stochastic Landau-Lifshitz-Gilbert equation^6^. In the Supplementary section, we present results of simulations to show that if *V*_0_ = 0.45 V, then switching is accomplished in 1.3 ns and the dynamic error probability associated with incorrect switching is less than 10^−8^ in every gate operation if we keep the voltage on for 1.3 ns. Therefore, the gate can work at a clock frequency of ~1/1.3 ns > 0.75 GHz with an error probability < 10^−8^. Stated succinctly, the probability of the output voltage being low when both inputs are high is > 99.999999% and the probability of it being low when either input is low is < 10^−8^. In other words, the NAND gate works with > 99.999999% fidelity. This is unimpressive for transistor-based volatile logic, but it is remarkable for non-volatile magnetic logic gates, which typically have very high error probabilities^6^,^7^,^8^. This degree of error-resilience may be sufficient for use in stochastic logic architectures^30^.

## Author Contributions

A.K.B. carried out the stochastic Landau-Lifshitz-Gilbert simulations to evaluate the error probability. S.B. and J.A. came up with the idea and verified the simulation results. All authors contributed to writing the paper.

## Supplementary Material

Supplementary Information Supplementary material

## Figures and Tables

**Figure 1:**
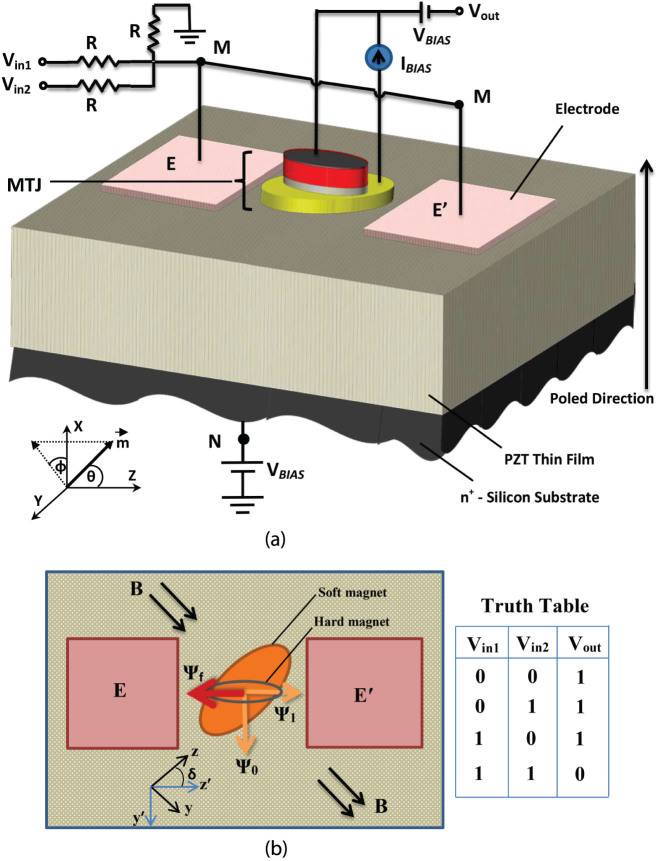
Structure of a NAND gate. (a) The PZT film has a thickness of ~100 nm and is deposited on a conducting n^+^-Si substrate. It is poled with an electric field in the direction shown. The distance between the electrodes is 100 nm and the electrode lateral dimensions are also of the same order. (b) The fixed magnetization orientation of the top (hard) magnet is denoted by Ψ*_f_*, and the two stable magnetization orientations of the bottom (soft) magnet are denoted by Ψ_0_ and Ψ_1_. The MTJ resistance is high when the soft magnet's magnetization is aligned along Ψ_1_. The MTJ resistance is (ideally) a factor of 2 lower when the soft magnet's magnetization is aligned along Ψ_0_. The slanted ellipse is the footprint of the soft magnet and the horizontal ellipse is the footprint of the hard magnet. The black double arrows show the direction of the permanent magnetic field.

**Figure 2:**
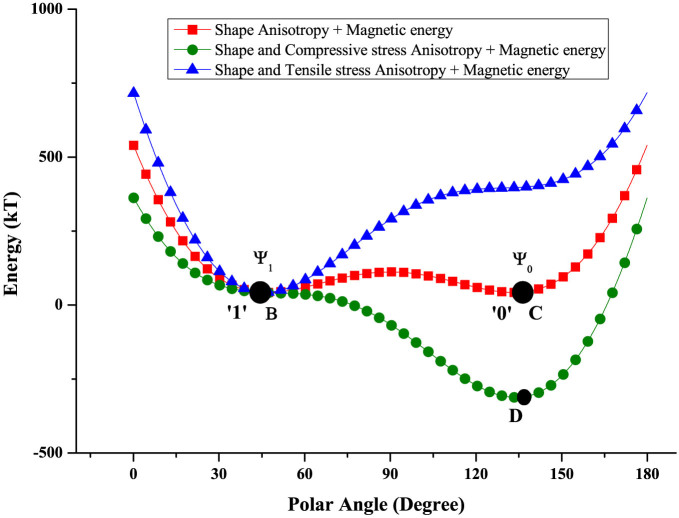
Potential energy profiles of the magnetostrictive layer in Fig. 1 as a function of its magnetization orientation. Energy plot as a function of polar angle (*θ*) of the magnetization vector, where the red line is for the unstressed magnet, the green line is for the compressively stressed magnet (−30 MPa), and the blue line is for the expansively stressed magnet (+30 MPa). The voltage levels between *M* and *N* that generate these stresses are ±112.5 mV.

**Figure 3:**
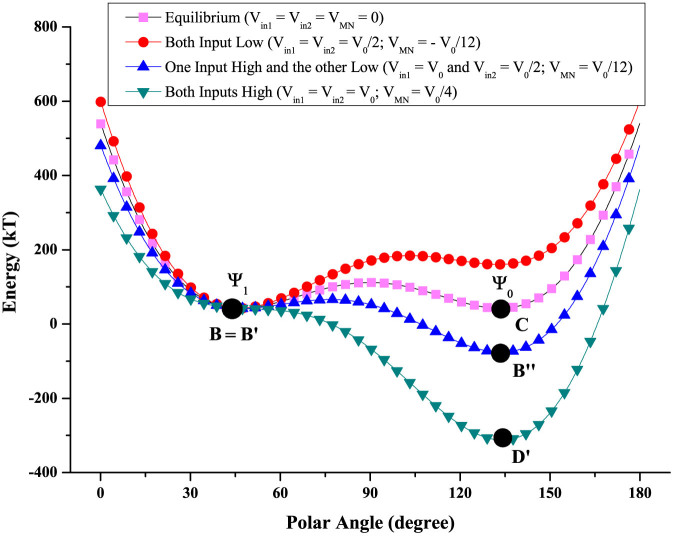
Potential energy profiles of the magnetostrictive layer in Fig. 1 for different logic inputs. Energy plot as a function of polar angle (*θ*) of the magnetization vector. The RESET operation brings the magnetization to state *B* where the magnetization is oriented along Ψ_1_ and the MTJ resistance is high. During logic operation, when both inputs are *low*, the magnet is under small tensile stress (+10 MPa) and the global energy minimum shifts slightly to *B*′ (*B* ≈ *B*′). Hence, the magnetization vector remains oriented very close to Ψ_1_ and the MTJ resistance remains high. If either input is *low*, the magnet is under small compressive stress (−10 MPa) and the global energy minimum moves to *B*″. However, there is an energy *barrier* of 23.63 kT separating *B*′ and *B*″, which cannot be transcended at room temperature. Consequently, the magnetization remains stuck at the local minimum near *B*′ and the MTJ resistance remains high. When both inputs are *high*, the magnet experiences high compressive stress (+30 MPa), which makes the energy profile monostable with a single energy minimum at *D*′ and no local minimum where the system can be trapped. Therefore, the system migrates to *D*′, the magnetization vector orients close to Ψ_0_, and the MTJ resistance goes low.

**Figure 4:**
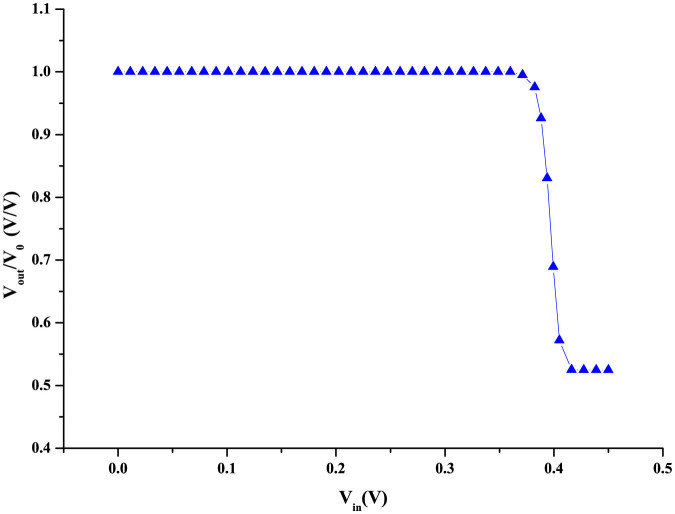
Transfer characteristic in the inverter mode. Shorting the two inputs of a NAND gate makes it an inverter. Plot of *V_out_* versus *V_in_* of the inverter at room temperature, where the *V_out_* values have been thermally averaged.
