# Supplementary material for: An error-resilient non-volatile magneto-elastic universal logic gate with ultralow energy-delay product
Source: Sci Rep. 2014 Dec 23;4:7553. doi: 10.1038/srep07553 (PMC4274515; doi:10.1038/srep07553)
Supplement: Supplementary Information — Supplementary material [file srep07553-s1.pdf]

Supplementary Information

An error-resilient non-volatile magneto-elastic universal logic gate with ultralow energy-delay product

Ayan Kumar Biswas[1], Jayasimha Atulasimha[2] and Supriyo Bandyopadhyay[1]

Email: {biswasak, jatulasimha, sbandy}@vcu.edu

[1]Dept. of Electrical and Computer Engg., [2]Dept. of Mechanical and Nuclear Engg.

Virginia Commonwealth University, Richmond, VA 23284, USA

November 3, 2014

In this accompanying supplementary material, we elucidate gate operation and concatenation, choice of the voltage level V_0, the stochastic Landau-Lifshitz-Gilbert simulations, and calculations of the transfer characteristic and energy dissipation in a gate operation.

Gate operation

To understand how the RESET, logic and the concatenation schemes work, consider Fig. S1. The nodes M and N represent the same nodes as in Fig. 1(a) of the main paper and V_{MN} is the voltage drop between these nodes. Therefore, V_{MN} is the voltage drop across the piezoelectric layer that generates strain in the magnetostrictive layer (soft layer of the MTJ) and makes its magnetization rotate. Note that V_{MN} alone determines the MTJ resistance. As established by the energy profiles in the main paper, when V_{MN} is either negative or positive but small, the hard and soft layers of the MTJ remain magnetized in anti-parallel directions and the MTJ resistance remains high. This high resistance is denoted by R_0. When V_{MN} is positive and sufficiently large in magnitude, the magnetizations of the hard and soft layers become mutually perpendicular and the MTJ resistance drops by a factor of 2 to become $R_0/2$.

Since the ratio R_{high}/R_{low} is 2:1, logic '1' must be encoded in some voltage level V_0 and logic '0' in voltage level $V_0/2$. This is needed because the logic levels at the output are determined solely by the MTJ resistance.

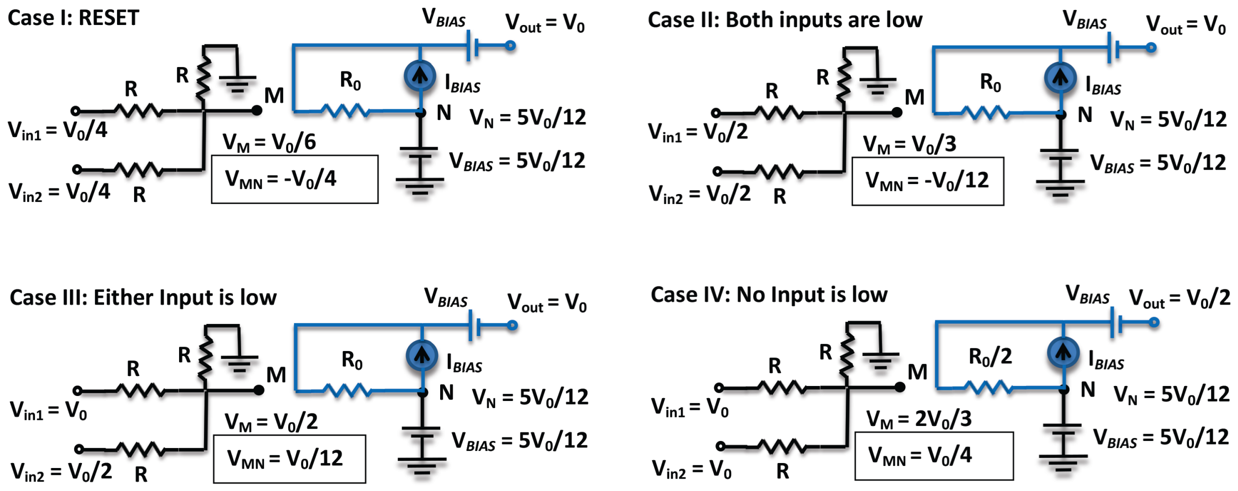

Figure S1: **Logic operations**

Let us consider the RESET operation that is supposed to leave the MTJ resistance in the high state R_0 (Case I in Fig. S1). The input voltages are set to $V_0/4$. The voltage at node M is then found by superposition and it is $V_0/6$. Since the bias voltage V_{BIAS} is set to $5V_0/12$, the voltage at node N is always fixed at $5V_0/12$. Therefore, V_{MN}, which is the voltage drop across the PZT thin film, becomes $-V_0/4$. This negative voltage generates *tensile* stress in the magnetostrictive layer and leaves its magnetization pointing anti-parallel to that of the the hard (SAF) layer of the MTJ (close to Ψ_1). Therefore, the MTJ resistance R_{MTJ} is left high at R_0 by the RESET step.

Note that the voltage drop between the bottom (soft) layer of the MTJ and node N is almost zero since the metallic layer shorts out the electric field underneath it in the PZT [S1]. Therefore, by applying Kirchoff's voltage law in the output loop, we find that

$$V_{out} = -V_{BIAS} + I_{BIAS}R_{MTJ} + V_{BIAS} = V_0\frac{R_{MTJ}}{R_0}, \tag{S1}$$

since I_{BIAS} is set to V_0/R_0. Because $R_{MTJ} = R_0$ after the RESET stage, $V_{out} = V_0$.

Next consider the logic operation stage when both inputs are low (Case II). Since $V_{in1} = V_{in2} = V_0/2$, V_M is $V_0/3$ and thus V_{MN} is $-V_0/12$. This negative voltage once again generates tensile stress in the magnetostrictive magnet that leaves the magnetizations of the hard and soft layers of the MTJ anti-parallel and the MTJ resistance high. Therefore, from Equation (S1), $V_{out} = V_0$. In other words, when both inputs are bit '0', the output is bit '1'.

When one input is high and the other low (Case III), $V_{in1} = V_0/2$ and $V_{in2} = V_0$ (the case where $V_{in1} = V_0$ and $V_{in2} = V_0/2$ is completely equivalent). The voltage at node M, V_M, is now $V_0/2$, which makes $V_{MN} = V_0/12$. The stress in the magnetostrictive layer is now compressive, but not compressive enough to rotate its magnetization by overcoming the shape anisotropy energy barrier

of the elliptical magnet. Therefore, MTJ resistance remains high at R_0 and the output voltage remains high at V_0. In other words, when one input is bit '1' and the other is bit '0', the output is bit '1'.

When both inputs are high (Case IV), $V_{in1} = V_{in2} = V_0$. In that case, V_M changes to $2V_0/3$, and V_{MN} becomes $V_0/4$. The stress generated by this magnitude of V_{MN} in the magnetostrictive layer is very compressive and sufficient to overcome the shape anisotropy barrier. As a result, the magnetization of the soft magnetostrictive layer now rotates by $\sim 90°$, placing it approximately perpendicular to that of the hard layer. Therefore, the MTJ's resistance drops to $R_0/2$ and [from Equation (S1)] the output voltage drops to $V_0/2$. Thus, when both inputs are bit '1', the output is bit '0'.

Note that the output voltage levels encoding bits '1' and '0' are V_0 and $V_0/2$ which are also the input voltage levels encoding bits '1' and '0'. Therefore, the output of one stage can be directly fed to the next stage as input without requiring additional hardware for amplification or level shifting. That makes this construct *concatenable.*

Choice of voltage level V_0

In order to choose the value of V_0 (which ultimately determines the amount of dissipation, switching delay and energy-delay product), we have to ensure that compressive stress generated by $V_{MN} = V_0/4$ is sufficient to overcome the shape anisotropy barrier in the elliptical magnetostrictive layer and rotate its magnetization, but compressive stress generated by $V_{MN} = V_0/12$ is not. The amount of stress generated by a certain voltage, and the effective shape anisotropy barrier in the presence of the permanent magnetic field, depend on many parameters such as the strength of the magnetic field, the shape and size of the magnetostrictive layer, the electrode size and placements, the piezoelectric layer thickness, and the piezoelectric and magnetostrictive materials. For the choices we made, we found from stochastic Landau-Lifshitz-Gilbert simulations of magnetodynamics in the presence of room-temperature thermal noise [S2] that a compressive stress of 30 MPa rotates the magnetization with greater than 99.999999% probability (and switches the MTJ resistance from high to low) in the presence of room-temperature thermal fluctuations, while a compressive stress of 10 MPa has less than 10^{-8} probability of rotating the magnetization and switching the MTJ resistance. Therefore, $V_{MN} = V_0/4$ needs to generate a stress of -30 MPa (compressive strain is negative). The material chosen for the magnetostrictive material is Terfenol-D because of its large magnetostriction. From the Young's modulus of Terfenol-D, we calculated that the strain required to generate a stress of

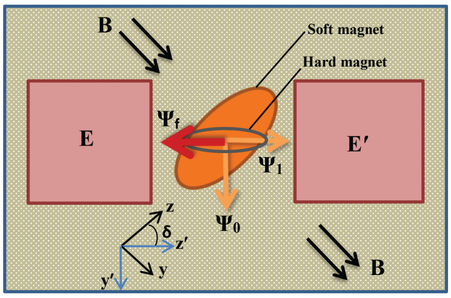

Truth Table

V_{in1}	V_{in2}	V_{out}
0	0	1
0	1	1
1	0	1
1	1	0

Figure S2: **The fixed magnetization orientation of the top (hard) magnet is denoted by Ψ_f, and the two stable magnetization orientations of the bottom (soft) magnet are denoted by Ψ_0 and Ψ_1. The MTJ resistance is high when the soft magnet's magnetization is aligned along Ψ_1. The MTJ resistance is (ideally) a factor of 2 lower when the soft magnet's magnetization is aligned along Ψ_0. The slanted ellipse is the footprint of the soft magnet and the horizontal ellipse is the footprint of the hard magnet. The black double arrows show the direction of the permanent magnetic field.**

-30 MPa is -3.75×10^{-4}. To generate this amount of strain, the strength of the electric field in the PZT between the shorted electrodes and the n$^+$-Si substrate should be 1.125 MV/m (interpolated from the results in Ref. [S1]). This value is well below the breakdown field of PZT. Since the PZT layer thickness is 100 nm, the voltage V_{MN} needed to generate the strain of -3.75×10^{-4} will be 112.5 mV. Hence, $V_0 = 4V_{MN} = 0.45$ V.

Stochastic Landau-Lifshitz-Gilbert (LLG) simulations

The error probability associated with gate operation, the internal energy dissipated during switching, and the switching delay – all in the presence of room-temperature thermal noise – are calculated from the stochastic Landau-Lifshitz-Gilbert equation. We first write expressions for the various contributions to the potential energy of the magnetostrictive layer and then find the effective torques due to these contributions as well as the random torque due to thermal noise. These torques rotate the magnetization vector. The entire procedure is described next.

We reproduce Fig. 1(b) from the main paper and define our coordinate system such that the magnet's easy (major) axis lies along the z-axis and the in-plane hard (minor) axis lies along the y-axis (see also Fig. 1(a) in main paper). Application of a positive/negative voltage between the electrode pair and the conducting n$^+$-Si substrate generates biaxial strain leading to compression/expansion along the z′-axis and expansion/compression along the y′-axis [S1]. The latter two axes are the axes of Ψ_1 and Ψ_0. The angle between the z- and z′ axes is δ, which is therefore the angle between the major axes of the hard and soft elliptical magnets.

To derive general expressions for the instantaneous potential energies of the nanomagnet due

to shape-anisotropy, stress-anisotropy and the static magnetic field, we used the primed axes of reference (x′, y′, z′) and represented the magnetization orientation of the single-domain magnetostrictive magnet in spherical coordinates with θ' representing the polar angle and ϕ' representing the azimuthal angle. The magnitude of the magnetization is invariant in time and space owing to the macrospin assumption.

Using the rotated coordinate system (see Fig. S2), the shape anisotropy energy of the nanomagnet $E_{sh}(t)$ can be written as,

$$\begin{aligned}
E_{sh}(t) &= E_{s1}(t)\sin^2\theta'(t) + E_{s2}(t)\sin 2\theta'(t) \\
&+ \frac{\mu_0}{2}\Omega M_s^2(N_{d-yy}\sin^2\delta + N_{d-zz}\cos^2\delta) \\
E_{s1}(t) &= \left(\frac{\mu_0}{2}\right)\Omega M_s^2\{N_{d-xx}\cos^2\phi'(t) + N_{d-yy}\sin^2\phi'(t)\cos^2\delta \\
&- N_{d-yy}\sin^2\delta + N_{d-zz}\sin^2\phi'(t)\sin^2\delta - N_{d-zz}\cos^2\delta\} \\
E_{s2}(t) &= \left(\frac{\mu_0}{4}\right)\Omega M_s^2\,(N_{d-zz} - N_{d-yy})\sin\phi'(t)\sin 2\delta,
\end{aligned} \tag{S2}$$

where $\theta'(t)$ and $\phi'(t)$ are respectively the instantaneous polar and azimuthal angles of the magnetization vector in the rotated frame, M_s is the saturation magnetization of the magnet, N_{d-xx}, N_{d-yy} and N_{d-zz} are the demagnetization factors that can be evaluated from the nanomagnet's dimensions [S3], μ_0 is the permeability of free space, and $\Omega = (\pi/4)abd$ is the nanomagnet's volume.

The potential energy due to the static magnetic flux density B applied along the in-plane hard axis is given by

$$E_m(t) = M_s\Omega B\left[\cos\theta'(t)\sin\delta - \sin\theta'(t)\sin\phi'(t)\cos\delta\right]. \tag{S3}$$

The stress anisotropy energy is given by

$$E_{str}(t) = -\frac{3}{2}\lambda_s\epsilon(t)Y\Omega\cos^2\theta'(t), \tag{S4}$$

where λ_s is the magnetostriction coefficient, Y is the Young's modulus, and $\epsilon(t)$ is the strain generated by the applied voltage V_{MN} at the instant of time t. We only consider the uniaxial strain along the line joining the two electrodes, but the strain is actually biaxial resulting in tension/compression along that line and compression/tension along the perpendicular direction. The torques due to these two components *add*. Therefore, we *underestimate* the stress anisotropy energy, which makes all our figures *conservative.*

We neglect any contribution due to the dipolar interaction of the hard magnet since the use of

the synthetic anti-ferromagnet makes it negligible.

The total potential energy of the nanomagnet at any instant of time t is therefore

$$E(t) = E\left(\theta'(t), \phi'(t)\right) = E_{sh}(t) + E_m(t) + E_{str}(t). \tag{S5}$$

The above result is used to plot the energy profiles in the main paper as a function of θ for $\phi = 90^\circ$ under various scenarios.

We follow the standard procedure to derive the time evolution of the polar and azimuthal angles of the magnetization vector in the rotated coordinate frame under the actions of the torques due to shape anisotropy, stress anisotropy, magnetic field and thermal noise.

The torque that rotates the magnetization of the shape-anisotropic magnet in the presence of stress can be written as

$$\begin{aligned}
\tau_{\mathbf{ss}}(t) &= -\mathbf{m}(t) \times \left(\frac{\partial E}{\partial \theta'(t)} \hat{\boldsymbol{\theta}} + \frac{1}{\sin\theta'(t)} \frac{\partial E}{\partial \phi'(t)} \hat{\boldsymbol{\phi}} \right) \\
&= \{E_{\phi 1}(t) \sin\theta'(t) + E_{\phi 2}(t) \cos\theta'(t) \\
&- M_s \Omega B \cos\delta \cos\phi'(t)\} \hat{\boldsymbol{\theta}} \\
&- \{E_{s1}(t) \sin 2\theta'(t) + 2E_{s2}(t) \cos 2\theta'(t) \\
&- M_s \Omega B (\cos\delta \sin\phi'(t) \cos\theta'(t) + \sin\delta \sin\theta'(t)) \\
&+ (3/2)\lambda_s \epsilon(t) Y \Omega \sin 2\theta'(t)\} \hat{\boldsymbol{\phi}},
\end{aligned} \tag{S6}$$

where $\mathbf{m}(t)$ is the normalized magnetization vector, quantities with carets are unit vectors in the original frame of reference, and

$$\begin{aligned}
E_{\phi 1}(t) &= \frac{\mu_0}{2} M_s^2 \Omega \{ \left(N_{d-yy} \cos^2\delta + N_{d-zz} \sin^2\delta \right) \sin 2\phi'(t) - N_{d-xx} \sin 2\phi'(t) \} \\
E_{\phi 2}(t) &= \frac{\mu_0}{2} M_s^2 \Omega \left(N_{d-zz} - N_{d-yy} \right) \sin 2\delta \cos\phi'(t).
\end{aligned}$$

At non-zero temperatures, thermal noise generates a random magnetic field $\mathbf{h}(t)$ with Cartesian components $(h_x(t), h_y(t), h_z(t))$ that produces a random thermal torque which can be expressed as [S2]

$$\tau_{\mathbf{th}}(t) = \mu_0 M_s \Omega \mathbf{m}(t) \times \mathbf{h}(t) = -\mu_0 M_s \Omega \left[h_\phi(t) \hat{\boldsymbol{\theta}} - h_\theta(t) \hat{\boldsymbol{\phi}} \right],$$

where

$$
\begin{aligned}
h_\theta(t) &= h_x(t)\cos\theta'(t)\cos\phi'(t) + h_y(t)cos\theta'(t)\sin\phi'(t) - h_z(t)sin\theta'(t) \\
h_\phi(t) &= -h_x(t)\sin\phi'(t) + h_y(t)cos\phi'(t).
\end{aligned} \tag{S7}
$$

In order to find the temporal evolution of the magnetization vector under the vector sum of the different torques mentioned above, we solve the stochastic Landau-Lifshitz-Gilbert (LLG) equation:

$$
\frac{d\mathbf{m}(t)}{dt} - \alpha\left[\mathbf{m}(t)\times\frac{d\mathbf{m}(t)}{dt}\right] = \frac{-|\gamma|}{\mu_0 M_s\Omega}\left(\tau_{\mathbf{ss}}(t) + \tau_{\mathbf{th}}(t)\right) \tag{S8}
$$

From the above equation, we can derive two coupled equations for the temporal evolution of the polar and azimuthal angles of the magnetization vector:

$$
\begin{aligned}
\frac{d\theta'(t)}{dt} &= -\frac{|\gamma|}{(1+\alpha^2)\mu_0 M_s\Omega}\{E_{\phi1}(t)\sin\theta'(t) + E_{\phi2}(t)\cos\theta'(t) \\
&- M_s\Omega B\cos\delta\cos\phi'(t) - \mu_0 M_s\Omega h_\phi(t) \\
&+ \alpha\{E_{s1}(t)\sin 2\theta'(t) - \mu_0 M_s\Omega h_\theta(t) \\
&+ 2E_{s2}(t)\cos 2\theta'(t) + (3/2)\lambda_s\epsilon(t)Y\Omega\sin 2\theta'(t) \\
&- M_s\Omega B(\cos\delta\sin\phi'(t)\cos\theta'(t) + \sin\theta'(t)\sin\delta)\}\}
\end{aligned} \tag{S9}
$$

$$
\begin{aligned}
\frac{d\phi'(t)}{dt} &= \frac{|\gamma|}{\sin\theta'(t)(1+\alpha^2)\mu_0 M_s\Omega}\{E_{s1}(t)\sin 2\theta'(t) \\
&+ 2E_{s2}(t)\cos 2\theta'(t) + (3/2)\lambda_s\epsilon(t)Y\Omega\sin 2\theta'(t) \\
&- M_s\Omega B(\cos\delta\sin\phi'(t)\cos\theta'(t) + \sin\delta\sin\theta'(t)) \\
&- \mu_0 M_s\Omega h_\theta(t) - \alpha(E_{\phi1}(t)\sin\theta'(t) + E_{\phi2}(t)\cos\theta'(t) \\
&- M_s\Omega B\cos\delta\cos\phi'(t) - \mu_0 M_s\Omega h_\phi(t))\}.
\end{aligned} \tag{S10}
$$

Solutions of these two equations yield the magnetization orientation $(\theta'(t), \phi'(t))$ at any instant of time t. Since the thermal torque is random, the solution procedure involves generating switching trajectories by starting each trajectory with an initial value of (θ', ϕ') and finding the values of these angles at any other time by running a simulation using a time step of $\Delta\text{t} = 1$ ps and for a sufficiently long duration. At each time step, the random thermal torque is generated stochastically. The time step is equal to the inverse of the maximum attempt frequency of demagnetization due to thermal noise in nanomagnets [S4]. The duration of the simulation is always sufficiently long to ensure that the final results are independent of this duration, and they are also verified to be independent of

the time step.

The permanent magnetic field ($B = 0.1305$ T) applied along the +y- direction (hard axis of the magnet) makes the two stable states of the soft magnet's magnetization align along Ψ_1 ($\theta = \theta_1 =$ 46.8°) and Ψ_0 ($\theta = \theta_0 =$ 133.2°) leaving a separation angle γ of 86.3° (Fig. S2) between them. Thermal noise however will make the magnetization of the soft magnet fluctuate around these two orientations and in order to determine the thermal distribution around Ψ_1 (which is where the RESET operation leaves the magnetization at), we solve the last two equations in the absence of any stress by starting with the initial state $\theta = 46.8°$ and $\phi = 90°$ and obtaining the final values of θ and ϕ by running the simulation for a long time. This process is repeated for 100 million switching trajectories. A histogram is then generated from these 100 million switching trajectories for the final values of θ and ϕ, which yields the thermal distribution around Ψ_1.

To study the switching dynamics under the influence of stress, we generate 100 million switching trajectories in the stressed state of the magnet by solving Equations (S9) and (S10), again using a time step of 1 ps. This time the initial magnetization orientation for each of the 10^8 trajectories is chosen from the thermal distributions generated in the previous step with the appropriate weightage since the RESET step always leaves the magnetization around state Ψ_1. The simulation is continued for 1.5 ns. We find that when the stress is either tensile (+10 MPa corresponding to $V_{MN} = -V_0/12$), or compressive but weak (-10 MPa corresponding to $V_{MN} = V_0/12$), the magnetization's polar angle returns to within 4° of Ψ_1 ($\theta = \theta_1 = 46.8°$) in 1.3 ns or less for every one of the 10^8 trajectories. After 1.3 ns, the stress is removed abruptly and the simulation is continued for an additional 0.2 ns to ensure that the final state does not change. It did not change for any of the 10^8 trajectories. This procedure tells us that when the inputs to the logic gate are both low, or one is high and the other is low, the magnetization of the soft layer of the MTJ does not rotate and the MTJ resistance remains high with >99.999999% probability. This fulfills the requirements of the NAND gate with >99.999999% probability.

In the case of one input low and one input high, what prevents rotation from Ψ_1 to Ψ_0 is the energy barrier of 23.63 kT between these two states as discussed in the main paper. This barrier is high enough to reduce the switching probability to below 10^{-8}.

When both inputs are high, a compressive stress of -30 MPa is generated in the magnetostrictive magnet. Once again, we pick the initial orientations of the magnetization from the thermal distribution around Ψ_1 which is where the RESET step leaves the magnet at, and generate 100 million switching trajectories as before. This time θ approaches within 4° of final state Ψ_0 ($\theta = \theta_0$ = 133.2°) in 1.3 ns or less. We continue the simulation for an additional 0.2 ns to confirm that once

the magnetization reaches the vicinity of Ψ_0, it settles around that orientation and does not return to the neighborhood of the initial orientation Ψ_1. We repeated this procedure for 10^8 times and found that every single switching trajectory behaved in the above manner. Therefore, we conclude that when the inputs to the logic gate are both high, the magnetization of the soft layer of the MTJ does rotate and the MTJ resistance goes low with >99.999999% probability. That fulfills the remaining requirement of the NAND gate with >99.999999% probability.

Transfer characteristics of NAND gate

When the two inputs of a NAND gate are shorted, it behaves like a NOT gate (inverter). If the input voltage to the inverter is between $V_0/12$ and $V_0/4$ (i.e. the compressive stress is between -10 MPa and -30 MPa), the energy profile becomes such that the magnetization of the soft layer may rotate to an intermediate state between Ψ_0 and Ψ_1 and fluctuate around that orientation because of thermal noise. We can time-average over the fluctuations to determine the 'steady-state' mean orientation at that input voltage V_{in} and thence calculate R_{MTJ} and $V_{out} = V_0 R_{MTJ}/R_0$. In order to do this, we calculate the stress generated by the V_{MN} corresponding to the input and run the stochastic Landau-Lifshitz-Gilbert simulation to determine the steady-state magnetization orientation and V_{out}. The purpose of this exercise is to find the transfer characteristic V_{out} versus V_{in}. This characteristic is shown in the main paper and shows a sharp transition. The transition range is from 0.38 V to 0.41 V (a range of 0.03 V) of input voltage whereas the logic levels are 0.225 V and 0.45 V. This portends excellent logic level restoration capability. In the high state, the input voltage can drift down by 0.04 volts and still produce the correct output state, while in the low state, the input voltage can drift up by 0.155 volts and still produce the correct output state.

Energy Dissipation

There are four avenues for energy dissipation in the proposed universal NAND gate: internal dissipation due to Gilbert damping that occurs while the magnetostrictive layer's magnetization switches (rotates), energy $C\left(V_{MN}\right)^2$ dissipated in turning on/off the potential $V_{MN} = \pm V_0/4$ (= 112.5 mV) abruptly or non-adiabatically during the RESET stage or logic operation stage (where C is the capacitance between the shorted pair of electrodes and the n^+-Si substrate), the energies dissipated in the resistors R, and the maximum energy V_0^2/R_{high} dissipated in the MTJ when the output is high (the energy dissipated when the output is low is $V_0^2/4R_{low} = V_0^2/2R_{high}$, which is 50% lower).

The energy dissipated due to Gilbert damping in a magnet is given by [S5, S6]

$$E_{d\_gd} = \int_0^{t_s} P_{d\_gd}(t)dt, \tag{S11}$$

where t_s is the switching delay (counted between the time the magnetization leaves the vicinity of Ψ_1 and arrives within 4° polar angle of Ψ_0) and $P_{d\_gd}(t)$ is the power dissipation and can be expressed as

$$P_{d\_gd}(t) = \frac{\alpha\gamma}{(1+\alpha^2)\mu_0 M_s \Omega} |\tau_{\mathbf{eff}}(t)|^2, \tag{S12}$$

where $\tau_{\mathbf{eff}}(t)$ is the torque due to shape anisotropy, stress anisotropy and the torque due to magnetic field (the thermal torque does not dissipate energy). The energy dissipation is obviously different for different switching trajectories, and we have found that the mean dissipation is 316 kT at room temperature. This calculation overestimates the energy dissipation slightly, but that only makes our figures conservative.

The next component is the $C\,(V_{MN})^2$ dissipation. We have electrodes of dimensions 100 nm $\times$ 100 nm and the thickness of the PZT layer is 100 nm. Thus, the capacitance between either electrode and the silicon substrate is C = 0.88fF, assuming that the relative dielectric constant of PZT is 1000. The voltage $V_{MN} = \pm V_0/4$ (= 112.5 mV). Since we have a *pair* of electrodes, the dissipation will be roughly twice $(1/2)C\,(V_{MN})^2$. We calculated that value as 2688 kT.

The dissipation in the resistance R can be negligible as we can make this resistance arbitrarily high.

Finally, we have to calculate the maximum energy dissipation due to the bias current flowing through the MTJ stack during a switching action. Since the bias current is flowing continuously, it results in *standby* energy dissipation which would be unacceptable in certain applications where the circuit is mostly dormant and wakes up to perform an operation infrequently (e.g. cell phones that wake up and perform a function only when a call or message is received). However, there are many applications where the circuit is constantly busy and seldom, if ever, in a standby mode (e.g. medical applications where the implanted device constantly monitors and processes signals). For such applications, standby dissipation is a not a serious concern.

In order to calculate this energy dissipation, let us assume that for the sake of adequate noise margin, the bias current I_{BIAS} can be no less than 1 pA. This restricts R_{high} to V_0/I_{BIAS} = 0.45×10^{12} ohms. The MTJ's resistance will increase super-linearly (almost exponentially) with the spacer layer thickness since current flows by tunneling through this layer, so the above resistance is

not difficult to achieve. The resulting maximum energy dissipation $V_0^2 t_s/R_{low}$ is 0.28 kT, which is negligible. Consequently, in a gate operation, the maximum energy dissipation is 3004 kT, which is comparable to that of low-power CMOS based NAND gates [S7], but the latter is *volatile* while the present gate is not. The energy dissipation is almost two orders of magnitude smaller than that in other magnetic non-volatile logic gates [S7].

References

[1] Cui, J. et al. A method to control magnetism in individual strain-mediated magnetoelectric islands. *Appl. Phys. Lett.* **103**, 232905 (2013).

[2] Roy, K., Bandyopadhyay, S. & Atulasimha, J. Energy dissipation and switching delay in stress-induced switching of multiferroic nanomagnets in the presence of thermal fluctuations. *J. Appl. Phys.* **112**, 023914 (2012).

[3] Chikazumi, S. *Physics of Magnetism* (Wiley New York, 1964).

[4] Gaunt, P. The frequency constant for thermal activitation of a ferromagnetic domain wall. *J. Appl. Phys.* **48**, 3470 (1977).

[5] Behin-Aein, B., Datta, D., Salahuddin, S. & Datta S., Proposal for an all-spin logic device with built-in memeory. *Nature Nanotech.* **5**, 266 - 269 (2010).

[6] Sun, Z. Z. & Wang, X. R., Fast magnetization switching of Stoner particles: A nonlinear dynamics picture. *Phys. Rev. B.* **71**, 174430 (2005).

[7] Nikonov, D. E. & Young, I. A., Overview of beyond CMOS devices and uniform methodology for their benchmarking. *Proc. IEEE*, **101**, 2498 (2013).
